# Psychotic symptoms in acromegaly

**DOI:** 10.4103/0019-5545.46078

**Published:** 2005

**Authors:** Denzil Pinto, A.T. Safeekh, Mohit Trivedi

**Affiliations:** * Associate Professor Department of Psychiatry, Father Muller Medical College, Kankanady, Mangalore, Karnataka 575002; ** Assistant Professor Department of Psychiatry, Father Muller Medical College, Kankanady, Mangalore, Karnataka 575002; *** PG Resident Department of Psychiatry, Father Muller Medical College, Kankanady, Mangalore, Karnataka 575002

**Keywords:** Schneiderian first-rank symptoms, psychotic symptoms, acromegaly

## Abstract

Various psychiatric symptoms have been reported in patients with acromegaly. Most of them are personality changes characterized by lack of initiative and spontaneity. There are few case reports of the presence of auditory and visual hallucinations, and delusions in patients with acromegaly. We report a patient with acromegaly who had psychotic symptoms including Schneiderian first-rank symptoms.

## INTRODUCTION

The relationship between endocrinology and psychiatry has always been of immense interest to psychiatrists as the presence of psychiatric symptoms in various endocrine disorders has been well demonstrated. The psychological accompaniments of acromegaly do not appear to have been studied systematically.^1^ Spence reported a case of acromegaly with persecutory delusions, visual and auditory hallucinations with depression.^2^ We report a case in which psychotic symptoms including Schneiderian first-rank symptoms were seen in a patient with acromegaly.

## THE CASE

A 45-year-old unmarried fisherman from the lower socioeconomic class presented with delusions of reference and persecution, second- and third-person auditory hallucinations and hallucinatory voices giving a running commentary of his actions. The onset of symptoms was insidious and of 6 months' duration, without any precipitating psychosocial factors. He was well oriented with intact attention, concentration and memory. He had total absence of insight. The patient reported decreased work performance, social withdrawal and sleep disturbance. There was no history of depressive mood or deliberate self-harm during the past 6 months and there was no history of substance use. There was no history of sweating, headache or visual disturbances.

The patient had been treated in another hospital for fearfulness, restlessness and persecutory ideas 13 years ago. A diagnosis of acute and transient psychotic disorder was made and the patient was put on haloperidol 10 mg, which was later tapered to 2 mg. The symptoms remitted within a month. There was no mention of acromegaly in the discharge summary. The patient failed to continue follow-up as he was busy with his work and was feeling well. His father had an alcohol dependence syndrome. His sister probably had depressive disorder for which she was on treatment including electroconvulsive therapy (ECT) and died at the age of 25 years; the exact cause of her death is not known. His brother, who had a psychotic illness characterized by hearing voices for which he was put on ayurvedic medicines, committed suicide by hanging himself within 2 weeks of the onset of symptoms.

On examination, the patient's pulse rate was 80/minute, and the blood pressure was 150/80 mmHg. His facial features were suggestive of acromegaly (enlargement of the supra-orbital ridges, macroglossia, coarse facial features, widening of the incisors and a prognathic mandible). He also had spade-like hands and feet. Kyphosis or features of hypogonadism were absent. Ophthalmic examination revealed a superior field deficit in the right eye and a nasal field deficit in the left eye. The fields of vision were checked using a Humphrey field analyser, which is an automated perimeter. It showed very high fixation losses and false-negative results. Hence, the visual field examination may not be reliable. The blood sugar and serum prolactin levels were within normal limits. The level of growth hormone was 6.5 ng/ml (normal 0.06–5.00 ng/ml). A CT scan revealed a moderately enhancing, enlarged pituitary gland and thickened skull. There was no involvement of the optic nerve. Calveral thickening, an enlarged sella tursica and a large frontal sinus were seen. The size of the enlarging tumour was 19 × 17 mm. There was no extrasellar extension, or extension into the sphenoid and cavernous sinuses. The optic chiasma was normal.

The patient was started on risperidone 2 mg and the dosage was gradually increased to 8 mg along with 4 mg of trihexyphenedyl and 2 mg of lorazepam. The psychotic symptoms completely remitted within a month. No specific treatment for acromegaly was given as the patient was non-compliant. The patient was followed up for a period of 6 months. There was no relapse of the psychotic symptoms or worsening of the symptoms of acromegaly.

## DISCUSSION

Acromegaly is a syndrome seen in adults due to excessive production of pituitary growth hormone as a result of adenoma or hyperplasia of the eosinophilic cells of the anterior pituitary gland. The clinical features of acromegaly include skeletal overgrowth affecting mainly the hands, feet, skull as well as kyphosis, hypertension, hypogonadism and diabetes mellitus.

Bleuler was the first to study psychiatric symptoms in patients with acromegaly.^3^ He described personality changes characterized by lack of initiative and spontaneity with brief periods of impulsive behaviour and, at times, cheerfulness and self-satisfaction. He also observed brief mood swings with spells of anxiety along with bradyphrenia, egocentricity and lack of concern. The presence of depressive symptoms was reported by Avery and Margo.^4^,^5^ Sivakumar and Williams reported a case of acromegaly with depression and pathological gambling.^6^ The presence of psychotic symptoms in a patient with acromegaly was described by Pye and Abbott in 1983.^7^ Their patient had delusions of reference, persecution, and visual and second-person auditory hallucinations. Spence reported the presence of delusions of persecution, visual and auditory hallucinations with depression in a patient with acromegaly.^2^

Abed *et al*. failed to prove any significant relationship between psychiatric morbidity and growth hormone levels. In general, the psychiatric morbidity in patients with acromegaly was comparable with morbidity in the general population.^8^ However, this study found that female patients with acromegaly had significantly greater psychiatric morbidity than males (11 of the 12 patients were females).

This case report describes the presence of psychotic symptoms including Schneiderian first-rank symptoms in the absence of visual hallucinations in a patient with acromegaly. However, it would be difficult to conclude that this patient's psychiatric symptoms were secondary to acromegaly because of the presence of a strong family history and the paucity of scientific data on the relationship between acromegaly and psychiatric morbidity.
